# Decreased Hematocrit-To-Viscosity Ratio and Increased Lactate Dehydrogenase Level in Patients with Sickle Cell Anemia and Recurrent Leg Ulcers

**DOI:** 10.1371/journal.pone.0079680

**Published:** 2013-11-04

**Authors:** Philippe Connes, Yann Lamarre, Marie-Dominique Hardy-Dessources, Nathalie Lemonne, Xavier Waltz, Danièle Mougenel, Martin Mukisi-Mukaza, Marie-Laure Lalanne-Mistrih, Vanessa Tarer, Benoit Tressières, Maryse Etienne-Julan, Marc Romana

**Affiliations:** 1 UMR Inserm 665, Université des Antilles et de la Guyane, Pointe-à-Pitre, Guadeloupe; 2 Laboratory of Excellence GR-Ex « The red cell : from genesis to death », PRES Sorbonne Paris Cité, Paris, France; 3 Laboratoire ACTES (EA 3596)/Département de Physiologie, Université des Antilles et de la Guyane, Pointe-à-Pitre, Guadeloupe; 4 Unité Transversale de la Drépanocytose, Centre Hospitalier et Universitaire de Pointe-à-Pitre, Pointe-à-Pitre, Guadeloupe; 5 CIC-EC 802 Inserm, Centre Hospitalier Universitaire de Pointe-à-Pitre, Pointe-à-Pitre, Guadeloupe; 6 Centre de référence maladies rares pour la drépanocytose aux Antilles-Guyane, Centre Hospitalier et Universitaire de Pointe-à-Pitre, Pointe-à-Pitre, Guadeloupe; Instituto de Higiene e Medicina Tropical, Portugal

## Abstract

Leg ulcer is a disabling complication in patients with sickle cell anemia (SCA) but the exact pathophysiological mechanisms are unknown. The aim of this study was to identify the hematological and hemorheological alterations associated with recurrent leg ulcers. Sixty-two SCA patients who never experienced leg ulcers (ULC-) and 13 SCA patients with a positive history of recurrent leg ulcers (ULC+) - but with no leg ulcers at the time of the study – were recruited. All patients were in steady state condition. Blood was sampled to perform hematological, biochemical (hemolytic markers) and hemorheological analyses (blood viscosity, red blood cell deformability and aggregation properties). The hematocrit-to-viscosity ratio (HVR), which reflects the red blood cell oxygen transport efficiency, was calculated for each subject. Patients from the ULC+ group were older than patients from the ULC- group. Anemia (red blood cell count, hematocrit and hemoglobin levels) was more pronounced in the ULC+ group. Lactate dehydrogenase level was higher in the ULC+ group than in the ULC- group. Neither blood viscosity, nor RBC aggregation properties differed between the two groups. HVR was lower and RBC deformability tended to be reduced in the ULC+ group. Our study confirmed increased hemolytic rate and anemia in SCA patients with leg ulcers recurrence. Furthermore, our data suggest that although systemic blood viscosity is not a major factor involved in the pathophysiology of this complication, decreased red blood cell oxygen transport efficiency (i.e., low hematocrit/viscosity ratio) may play a role.

## Introduction

Leg ulcers have been previously reported in 25-65% of adult sickle cell patients in Jamaica [1,2] but the incidence is much lower (2-2.5%) in North America [2,3]. Leg ulcers are often refractory to treatment and are prone to recurrence [1,2]. While the exact causes of recurrent leg ulcers are unknown [4–7], several studies support a role of hemolysis. Nolan et al [7] shown that patients with sickle cell anemia (SCA) and leg ulcers had lower hemoglobin level and higher levels of lactate dehydrogenase (LDH), bilirubin, aspartate transaminase and reticulocyte counts than SCA patients without leg ulcers. Similar observation was made by Taylor et al [8] who observed higher frequency of patients with a positive history of leg ulcers in the top quartile of serum LDH level in the NIH database and the Cooperative Study of Sickle Cell Disease database (CSSD). Cumming et al [9], in The Jamaican Cohort Study, reported that high serum LDH, with low socio-economic status and venous incompetence, were strong predictors of chronic leg ulceration in SCA patients. Indeed, leg ulcer is considered to belong to the hemolytic-endothelial dysfunction sub-phenotype [10], with chronic hemolysis being responsible for the decrease of nitric oxide (NO) bio-availability leading to impaired vasomotor tone and promoting the development of vasculopathy. 

Recently, a study reported that SCA patients with leg ulcers exhibited greater blood viscosity that patients without this complication, despite a lower hematocrit in the former group [4]. The authors hypothesized that the higher blood viscosity probably compromise tissue oxygenation and thus promote the development of leg ulcers [4]. Their hypothesis was partly supported by the lower hematocrit-to-viscosity ratio - an index which reflects the red blood cell oxygen transport efficiency [11] - in patients with leg ulcers. However, this finding is surprising since the greater hemolytic rate and the more pronounced anemia in SCA patients with leg ulcers should lead to a lower blood viscosity. To address this paradox, we compared the hematological, biochemical and hemorheological profiles of SCA patients with recurrent leg ulcers to patients who never had leg ulcers. Patients characterized by recurrent leg ulcers did not have this complication at the time of the study to avoid any acute inflammatory effects on blood rheology [12]. The aim of this study was to identify the hemorheological alterations associated with recurrent leg ulcers.

## Material and Methods

### Patients

The study is a part of the “Sickle cell hemorheology study” [13,14], which took place between May 2010 and December 2011. Ninety-seven adults with SCA regularly followed by the Sickle Cell Center at the Academic Hospital of Pointe-à-Pitre (Guadeloupe, French West Indies) were included in this study. However, 19 SCA patients were excluded from the analysis because they were under hydroxyurea therapy, which is known to modulate blood rheology and hematological parameters [14,15]. Three other SCA patients were also excluded because they had leg ulcers at the time of the study. Thus, 62 SCA patients who never experienced leg ulcers (ULC-) and 13 SCA patients with a positive history of recurrent leg ulcers (ULC+) - but with no leg ulcers at the time of the study - were included in the analyses. Age at which the first leg ulcer occurred, the mean duration and the mean frequency of leg ulcers episodes are given in the Table 1. All patients were in steady-state condition at the time of the study: no blood transfusions in the previous three months and absence of acute episodes (infection, vaso-occlusive crisis (VOC), acute chest syndrome (ACS), stroke, priapism) at least two months before inclusion into the study. 

**Table 1:** Comparison of the general characteristics and hematological parameters between SCA patients without (ULC-) and with (ULC+) leg ulcers.

	**ULC-**	**ULC+**
Sex (M/F)	26/36	4/9
Age (years)	31.8 ± 12.3	41.9 ± 12.6**
Age at which the first leg ulcer occurred (years)^$^	-	26.2 ± 10.5
Mean duration of leg ulcer episodes (months)		6.5 ± 10.0
Frequency of leg ulcers episodes (per year)		0.7 ± 0.4
Height (cm)	169 ± 9	171 ± 8
Weight (kg)	61.8 ± 9.9	61.0 ± 11.0
Body mass index (kg/m^2^)	21.6 ± 3.4	21.0 ± 4.3
HbF (%)	8.2 ± 5.6	9.4 ± 7.3
alpha-thalassemia (%)	45.2	46.2
RBC (10^12^/L)	3.0 ± 0.6	2.6 ± 0.5*
WBC (10^9^/L)	9.9 ± 2.3	10.4 ± 2.9
PLT (10^9^/L)	414 ± 132	373 ± 115
Hb (g/dL)	8.6 ± 1.2	7.8 ± 1.4*
Hct (%)	24.2 ± 3.5	21.7 ± 3.8*
MCV (fl)	82.7 ± 8.9	84.4 ± 6.5
MCHC (pg)	29.5 ± 3.4	30.1 ± 3.8
RET (%)	8.3 ± 3.1	8.6 ± 3.2
BIL mM	58.8 ± 46.0	51.3 ± 21.1
LDH (UI/L)	491 ± 156	591 ± 147*
AST (UI/L)	37 ± 12	43 ± 15
Hemolytic component (relative unit)	-0.10 ± 0.87	0.37 ± 0.97
CRP (mg/L)	6.45 ± 6.17	7.25 ± 6.94

Values represent mean ± SD. HbF, percent of foetal hemoglobin; RBC, red blood cell; WBC, white blood cell; PLT, platelet; Hb, hemoglobin concentration; Hct, hematocrit; MCV, mean corpuscular volume; MCH, mean corpuscular hemoglobin; RET, reticulocytes; BIL, bilirubin; LDH, lactate dehydrogenase; AST, aspartate aminotransferase; CRP, C-reactive protein level. Significant difference (*p < 0.05; **p < 0.01). ^$^No difference with the mean age of the ULC- group.

All patients were informed about the purpose and procedures of the study, and gave their written consent. The study was conducted in accordance with the guidelines set by the Declaration of Helsinki and was approved by the Regional Ethics Committee (CPP Sud/Ouest Outre Mer III, Bordeaux, France, registration number: 2010-A00244-35). 

### Genetic parameters

SCA diagnosis was made by isoelectrofocusing (Multiphor II™ System, GE HEALTH CARE, Buck, UK), citrate agar electrophoresis, and cation-exchange high performance liquid chromatography (VARIANT™, Bio-Rad Laboratories, Hercules, CA, USA), and was confirmed by DNA studies [16]. Polymerase Chain Reaction (Gap-PCR) was used to detect 6 common alpha-thalassemia deletions [17,18]. 

### Biological parameters

Blood samples were drawn after a 12-hrs overnight fasting, between 8:00 a.m. and 10:00 a.m., and were immediately used for analyses. 

Hemoglobin concentration (Hb), hematocrit (Hct), mean corpuscular volume (MCV), mean corpuscular hemoglobin (MCH), reticulocytes (RET), red blood cell (RBC), platelet (PLT) and white blood cell (WBC) counts were determined using hematology analyzer (Max M-Retic, Coulter, USA). Measurements of hemolytic markers (bilirubin, BIL; lactate dehydrogenase, LDH; aspartate aminotransferase, AST) and C-reactive protein (CRP) level were performed using standard biochemistry. A principal component analysis was used to derive a hemolytic component from the 4 hemolytic markers measured (i.e. BIL, LDH, AST and reticulocytes expressed in percentage). This standard statistical data reduction approach uses conventional clinical measurements to explain the maximum-shared variance among these indirect measures of hemolysis [19]. The hemolytic component has recently been demonstrated to reflect intravascular hemolysis assessed by measurements of the cell-free plasma hemoglobin [19].

Blood viscosity was measured after complete oxygenation of the blood, at native Hct, at 25°C and at a shear rate of 225 s^−1^ using a cone/plate viscometer (Brookfield DVII+ with CPE40 spindle, Brookfield Engineering Labs, Natick, MA) [20]. The hematocrit-to-viscosity ratio (HVR) was calculated as follows: Hct/blood viscosity at 225 s^-1^, with Hct determined by microcentrifugation techniques [11]. HVR reflects a balance between blood oxygen carrying capacity (i.e., hematocrit) and blood flow resistance (i.e., blood viscosity): the highest the HVR, the greatest the RBC oxygen transport effectiveness [11,21,22].. RBC deformability was determined at 37°C at 30 Pa by laser diffraction analysis (ecktacytometry), using the Laser-assisted Optical Rotational Cell Analyzer (LORCA, RR Mechatronics, Hoorn, The Netherlands). RBC aggregation was determined at 37°C via syllectometry, (i.e., laser backscatter versus time, using the LORCA) after adjustment of the Hct to 40%, and was reported as the aggregation index (AI). The RBC disaggregation threshold (Dis^th^), i.e., the minimal shear rate needed to prevent RBC aggregation or to break down existing RBC aggregates, was determined using a re-iteration procedure. The guidelines for international standardization in blood rheology techniques/measurements were strictly followed [20].

### Statistical analysis

Results are presented as means ± SD. Unpaired Student’s t-test and chi-square test were used for continuous covariates and for categorical covariates, respectively, to compare biological parameters between the ULC- and ULC+ groups. Principal component analysis was used to derive the hemolytic component. Significance level was defined as p < 0.05. Analyses were conducted using SPSS (v. 20, IBM SPSS Statistics, Chicago, IL). 

## Results and Discussion

### General, hematological and biochemical parameters (Table 1)

ULC+ patients were older than ULC- patients (p < 0.01), but the age at which the first leg ulcer occurred in the ULC+ group was not different from the mean age of the ULC- group. A predominance of females was observed in the ULC+ group, which contrasts with a recent study performed by Delaney et al [3] on a larger cohort. RBC count, Hb and Hct levels were lower in the ULC+ group compared to the ULC- group (p < 0.05). As already reported [7–9,23], this greater anemia was attributed to the greater hemolytic rate observed in the ULC+ group: although RET and BIL were not different between the two groups, AST tended to be increased (p < 0.1), LDH was higher (p < 0.05) and the hemolytic component tended to be higher (p < 0.1) in the ULC+ group compared to the ULC- group. The higher hemolytic rate is expected to decrease the amount of circulating NO and promote endothelial dysfunction [10]. Bowers et al [4] found increased level of sICAM-1 in SCA patients with leg ulcers, which supports increased activated state of the endothelial cells. The reason for the increased hemolytic rate and lower Hb level in SCA patients with a positive history of leg ulcers has been attributed to the lower frequency of alpha-thalassemia [23]. While we previously showed in our cohort of SCA adults that those with alpha-thalassemia had lower hemolytic rate and increased Hb level [14,24], we did not detect any difference in the frequency of alpha-thalassemia between ULC+ and ULC- patients, which is in agreement with another study [25]. These apparently conflicting results could be due to the small magnitude of the effect of alpha-thalassemia on leg ulcers [25]. 

### Hemorheological parameters (Table 2)

**Table 2:** Comparison of hemorheological parameters between SCA patients without (ULC-) and with (ULC+) leg ulcers.

	**ULC-**	**ULC+**
Blood viscosity (mPa.s^-1^)	6.1 ± 1.1	6.1 ± 0.9
HVR (a.u.)	4.1 ± 0.8	3.6 ± 0.5*
RBC deformability at 30 Pa	0.37 ± 0.09	0.32 ± 0.09
RBC aggregation index (%)	52 ± 10	55 ± 9
RBC disaggregation threshold (s^-1^)	289 ± 145	297 ± 147

Values represent mean ± SD. HVR, hematocrit-to-viscosity ratio; RBC, red blood cell. Significant difference (*p < 0.05).

Despite lower Hb and Hct levels observed in the ULC+ group, we detected no difference in blood viscosity between the two groups. This result may seem surprising but blood viscosity at high shear rate is also widely influenced by RBC deformability: poor deformable RBCs cannot easily align into the direction of the flow, which ultimately increases the flow resistance [26]. We detected a tendency for a lower RBC deformability in the ULC+ patients compared to the ULC- group (p < 0.1). It is therefore tempting to hypothesize that blood viscosity was not different between the two groups because RBC deformability of the ULC+ group was reduced by 16% compared to that of ULC- patients. The mechanism of the RBC deformability reduction in ULC+ patients remains unknown but this finding is in agreement with the results recently reported by Bartolucci et al [27] who demonstrated that the percent of dense dehydrated red blood cells (i.e. the more rigid) was greater in patients with leg ulcers compared to those without. In addition, the authors [27] reported significant relationships between the percent of dense RBCs and Hb level (r = -0.40; p < 0.001) and different hemolytic markers: LDH (r = 0.43; p < 0.001) and bilirubin (r = 0.30; p < 0.001). We found a significant negative relationship between the hemolytic component and RBC deformability (r = - 0.60; p < 0.001). This finding suggests that a lower RBC deformability is associated with higher rate of hemolysis: probably because rigid RBCs are more fragile. Surprisingly, Bowers et al [4] found higher blood viscosity in patients with leg ulcers despite lower Hb and Hct levels compared to patients without leg ulcers. However, the patients tested by Bowers et al [4] had ongoing leg ulcers at the time of the experiment, in contrast with our study, and local factors such as inflammation [28] could have affected blood viscosity. Nevertheless, in agreement with Bowers et al [4], the ULC+ group had a lower HVR than the ULC- group (p < 0.05). Because tissue oxygenation may depend on HVR [11,29], a low HVR could participate to the pathophysiology of this recurrent complication. 

In conclusion, our study confirms that patient with recurrent ulcers have a higher LDH and lower Hb than patients who never had ulcers. Furthermore, our data suggest that although systemic blood viscosity is not a major factor involved in the pathophysiology of this complication, decreased red blood cell oxygen transport efficiency (i.e., low hematocrit/viscosity ratio) may play a role. This study is a post-study analysis deriving from previous data [13,14]. The limited sample size and the fact that patients from the ULC+ group were older than patients from the ULC- group represent a major limitation. For example, it is unknown whether age may affect hemorheological parameters in SCA patients. Indeed, to strengthen our data, future studies will have to match patients with and without leg ulcers on age.
